# Supplementary material for: Chronic infections can generate SARS-CoV-2-like bursts of viral evolution without epistasis
Source: bioRxiv. 2024 Oct 7:2024.10.06.616878. Preprint. [Version 1] doi: 10.1101/2024.10.06.616878 (PMC11482859; doi:10.1101/2024.10.06.616878)
Supplement: Supplement 1 [file NIHPP2024.10.06.616878v1-supplement-1.pdf]

bioRxiv preprint doi: https://doi.org/10.1101/2024.10.06.616878; this version posted October 7, 2024. The copyright holder for this preprint (which was not certified by peer review) is the author/funder, who has granted bioRxiv a license to display the preprint in perpetuity. It is made available under aCC-BY 4.0 International license.

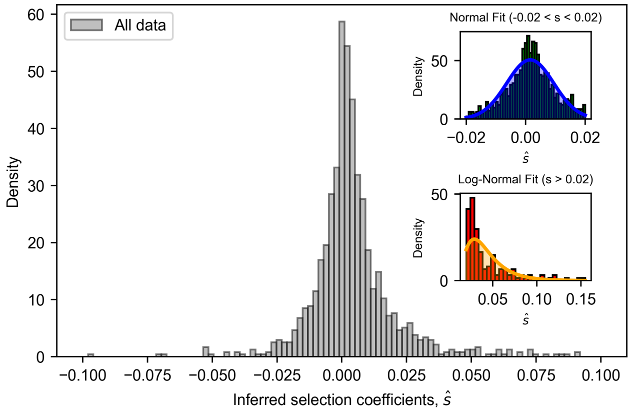

Supplementary Fig. 1. Inferred transmission effects of SARS-CoV-2 mutations. The main plot displays a histogram of selection coefficient values inferred from SARS-CoV-2 temporal genomic data[61]. The top-right inset plot shows the normal distribution fit for coefficient values considered neutral $(-0.02 < \hat{s} < 0.02)$; from this distribution, neutral mutation effects during within-host evolution were sampled. The bottom-right inset plot shows the log-normal distribution fit for values greater than 0.02, representing significantly beneficial mutations; from this distribution, beneficial mutation effects during within-host evolution were sampled.

bioRxiv preprint doi: https://doi.org/10.1101/2024.10.06.616878; this version posted October 7, 2024. The copyright holder for this preprint (which was not certified by peer review) is the author/funder, who has granted bioRxiv a license to display the preprint in perpetuity. It is made available under aCC-BY 4.0 International license.

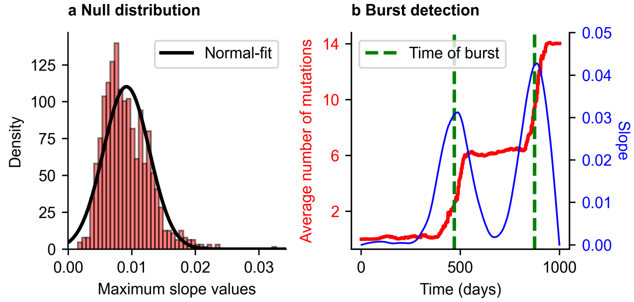

Supplementary Fig. 2. Detection of mutation bursts. a, Distribution of maximum slopes of accumulated mutation trajectories without chronic infection for acute generation time of $t_a = 4.0$ days and mutation rates: $\mu_B = 10^{-3}$ beneficial mutations per cycle and $\mu_N = 10^{-4}$ neutral mutations per cycle. **b,** For a simulation with chronic infection fraction $p_c = 10^{-4}$, the number of accumulated mutations averaged over an individual's population is shown in red. The blue curve indicates the smoothed slope time series with two peaks, detected by z-score time series change points and represented by the vertical green dashed lines. For smoothing using the Savitzky–Golay filter, we use parameters $w = 150$ and $p = 1$.

bioRxiv preprint doi: https://doi.org/10.1101/2024.10.06.616878; this version posted October 7, 2024. The copyright holder for this preprint (which was not certified by peer review) is the author/funder, who has granted bioRxiv a license to display the preprint in perpetuity. It is made available under aCC-BY 4.0 International license.

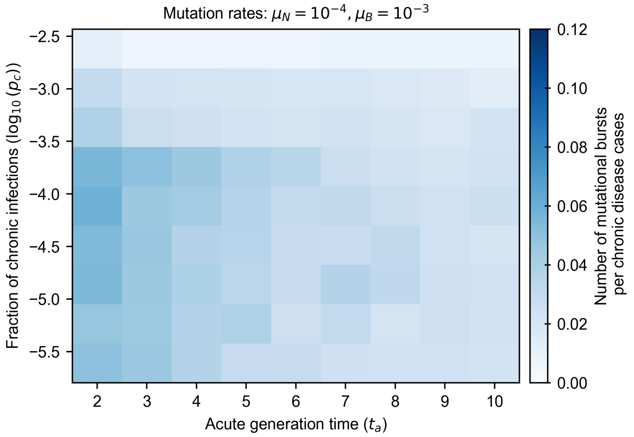

Supplementary Fig. 3. Number of mutational bursts per chronic disease case for beneficial mutation rate of 10^{-3} mutations/cycle. This figure is analogous to **Fig. 4** in the main text, but with a lower beneficial mutation rate.

bioRxiv preprint doi: https://doi.org/10.1101/2024.10.06.616878; this version posted October 7, 2024. The copyright holder for this preprint (which was not certified by peer review) is the author/funder, who has granted bioRxiv a license to display the preprint in perpetuity. It is made available under aCC-BY 4.0 International license.

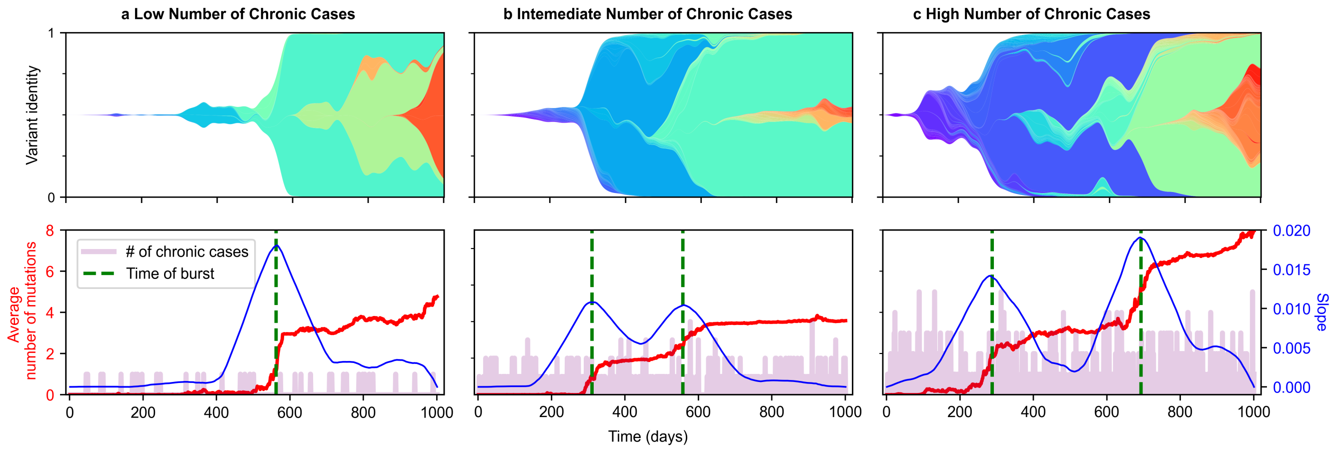

Supplementary Fig. 4. Dynamic evolution of the viral population under varying chronic infection probabilities. a, Low number of chronic cases, corresponding to a probability per transmission event of $p_c = 4 \times 10^{-4}$. **b** Intermediate number of chronic cases, with a probability per transmission event of $p_c = 3.7 \times 10^{-3}$. **c**, High number of chronic cases, resulting from a probability per transmission event of $p_c = 7.0 \times 10^{-3}$. For all simulations, we consider beneficial and neutral mutation rates $\mu_B = 10^{-4}$ mutations/cycle and $\mu_N = 10^{-4}$ mutations/cycle, respectively. Generation times are set at $t_a = 2$ for acute cases, while for chronic cases, they follow a log-normal distribution with a mean of $\mu_L = 150$ days and a standard deviation of $\sigma_L = 80$ days.
